# Toward Engineering the Mannose 6-Phosphate Elaboration Pathway in Plants for Enzyme Replacement Therapy of Lysosomal Storage Disorders

**DOI:** 10.3390/jcm8122190

**Published:** 2019-12-12

**Authors:** Ying Zeng, Xu He, Tatyana Danyukova, Sandra Pohl, Allison R. Kermode

**Affiliations:** 1 Department of Biological Sciences, Simon Fraser University, Burnaby, BC V5A1S6, Canada; yzeng@sfu.ca (Y.Z.); hhershy@gmail.com (X.H.); 2 Department of Osteology and Biomechanics, University Medical Center Hamburg-Eppendorf, 20246 Hamburg, Germany; t.danyukova@uke.de (T.D.); s.pohl@uke.de (S.P.)

**Keywords:** α-L-iduronidase (IDUA), mannose 6-phosphate (M6P), M6P elaboration machinery, plant-based platform, enzyme replacement therapy (ERT), transgenic plant, lysosomal enzyme

## Abstract

Mucopolysaccharidosis (MPS) I is a severe lysosomal storage disease caused by α-L-iduronidase (IDUA) deficiency, which results in accumulation of non-degraded glycosaminoglycans in lysosomes. Costly enzyme replacement therapy (ERT) is the conventional treatment for MPS I. Toward producing a more cost-effective and safe alternative to the commercial mammalian cell-based production systems, we have produced recombinant human IDUA in seeds of an *Arabidopsis* mutant to generate the enzyme in a biologically active and non-immunogenic form containing predominantly high mannose N-linked glycans. Recombinant enzyme in ERT is generally thought to require a mannose 6-phosphate (M6P) targeting signal for endocytosis into patient cells and for intracellular delivery to the lysosome. Toward effecting *in planta* phosphorylation, the human M6P elaboration machinery was successfully co-expressed along with the recombinant human IDUA using a single multi-gene construct. Uptake studies using purified putative M6P-IDUA generated *in planta* on cultured MPS I primary fibroblasts indicated that the endocytosed recombinant lysosomal enzyme led to substantial reduction of glycosaminoglycans. However, the efficiency of the putative M6P-IDUA in reducing glycosaminoglycan storage was comparable with the efficiency of the purified plant mannose-terminated IDUA, suggesting a poor *in planta* M6P-elaboration by the expressed machinery. Although the *in planta* M6P-tagging process efficiency would need to be improved, an exciting outcome of our work was that the plant-derived mannose-terminated IDUA yielded results comparable to those obtained with the commercial IDUA (Aldurazyme^®^ (Sanofi, Paris, France)), and a significant amount of the plant-IDUA is trafficked by a M6P receptor-independent pathway. Thus, a plant-based platform for generating lysosomal hydrolases may represent an alternative and cost-effective strategy to the conventional ERT, without the requirement for additional processing to create the M6P motif.

## 1. Introduction

Mucopolysaccharidosis (MPS) I is one of the over 70 lysosomal storage diseases that are caused by mutations in proteins critical for lysosomal function. The deficiency of α-L-iduronidase (IDUA) that underlies MPS I disease results in the accumulation of non-degraded glycosaminoglycans (GAGs) and causes multisystem pathology in a progressive manner [1]. Enzyme replacement therapy (ERT) is the conventional treatment for this genetic disease [2], but is costly, partly due to the traditional modes of production of recombinant IDUA.

Toward producing a more cost-effective and safe alternative to the commercial mammalian cell-based production systems, we have established an efficient plant-based recombinant protein production platform resulting in high-level synthesis of active human IDUA in seeds of the complex-glycan-deficient (cgl) mutant of *Arabidopsis thaliana*. The seed-based platform accumulates human IDUA at ~5.7% total soluble protein and the enzyme is generated in a non-immunogenic form containing predominantly high mannose N-linked glycans (~94%) [3,4,5,6]. Purified cgl-recombinant IDUA was used to determine the 3-D structure of the human protein along with the essential details of its catalytic mechanism [7].

The seed-derived IDUA possesses biological activities and kinetic properties that are very similar to the commercial IDUA (Aldurazyme^®^ (Sanofi, Paris, France)) used for ERT and derived from cultured Chinese hamster ovary (CHO) cells [6]. Our high-yielding *Arabidopsis* cgl seed line is thus a viable system for generating IDUA that is potentially suitable for treating patients with MPS I [6,8]. However, a parenterally-administered recombinant enzyme in ERT must have suitable targeting signals for endocytosis into patient cells and for intracellular delivery to the lysosome in order to be therapeutically efficacious. For most lysosomal enzymes, including IDUA, this generally requires the cellular recognition marker mannose 6-phosphate (M6P) onto the replacement protein [9,10]. Two Golgi-localized enzymes act sequentially in mammalian cells to elaborate M6P tags on lysosomal enzymes: the GlcNAc-1-phosphotransferase (PT) that adds UDP-GlcNAc to selected terminal mannose residues of the target enzyme’s high mannose N-glycans, and the N-acetylglucosamine-1-phosphodiester α-N-acetylglucosaminidase, also known as the ‘uncovering enzyme’ (UCE) that cleaves the GlcNAc residue to expose the M6P tag [11,12,13]. Importantly, the protein specificity underlying M6P elaboration rests with this first enzyme, which is localized to the *cis*-Golgi compartment. Thus the GlcNAc-1-phosphotransferase possesses the unique ability to distinguish the more than 70 lysosomal enzymes from the numerous non-lysosomal glycoproteins with identical Asn-linked glycans [14].

The PT is a 540-kDa hexameric enzyme complex consisting of α, β, and γ subunits; the functional complex is created by dimers of each of the subunit to form α_2_β_2_γ_2_ [15,16,17]. Both the α and β subunits are encoded by a single gene, *GNPTAB*, and the initial precursor protein of 1,256 amino acid residues is cleaved between D929 and K930 by the site-1 protease (S1P) [15,17,18]. This cleavage is a pre-requisite for the catalytic activity of the enzyme, and deficiency of S1P in mammalian cells results in non-functional PT enzymes [18,19]. Following cleavage, the α and β subunits are each anchored in the *cis*-Golgi apparatus by a single transmembrane domain. The soluble PT γ subunit (PT-γ) of 305 amino acids is encoded by a separate gene, *GNPTG* [20]. It contains a M6P receptor homology (MRH) domain, a protein domain whose function is to bind high-mannose-type N-glycans [21]. The γ subunit enhances the PT catalysis to a subset of the lysosomal hydrolases [12,22].

The UCE is a type I membrane-spanning glycoprotein of the trans-Golgi network (TGN); comprised of 515 amino acids, it contains a 25-amino acid signal peptide, a 24-amino acid propeptide, a luminal domain, a single transmembrane region, and a cytoplasmic tail [23,24,25]. Although it resides primarily in the TGN, it cycles between this compartment and the plasma membrane [26]. The UCE protein is synthesized as an inactive proenzyme; upon reaching the TGN, it is activated by the endoprotease furin, which cleaves an RARLPR↓D sequence to release the 24-amino acid propeptide [27]. As the final step to generating the M6P motif responsible for high affinity binding to M6P receptors, the UCE plays an essential role in lysosomal enzyme targeting.

Plants do not possess the enzymatic machinery to elaborate the M6P tag onto target proteins―the PT and UCE. Yet in previous work we have demonstrated that the purified plant-recombinant IDUA is amenable to sequential in vitro processing using soluble forms of the PT and UCE to add the M6P recognition marker [4,6].

The main strategy of the present report was to express the entire M6P elaborating human enzyme machinery together with IDUA in seeds to effect in vivo processing. Provided the endogenous recombinant plant IDUA is modified *in planta* by simultaneous synthesis of the human PT and UCE enzymes, the production platform would be particularly attractive since no downstream processing beyond M6P-IDUA purification would be required. Herein we detail our work on the expression of all the components of the M6P-human enzyme machinery in *Arabidopsis* cgl seeds that are already expressing IDUA. As a proof-of-principle, the human UCE protein was expressed as a soluble secreted protein in cgl *Arabidopsis* seeds. The purified plant-recombinant soluble UCE exhibited high enzymatic activity and, in vitro, the UCE was able to cleave the terminal GlcNAc residue from an artificial substrate and to generate the M6P onto a PT-processed plant recombinant IDUA.

A seed-specific promoter was used to drive expression, and sequences specifying a plant signal peptide replaced the human signal peptide sequences. Terminator and 3′-end sequences from the seed arcelin gene replaced the corresponding native sequences of the human genes. The three genes (PT-αβ, PT-γ, and UCE) were first expressed individually to determine their suitability and non-toxicity to plant growth and development, and to determine if the proteins would undergo the expected post-translational processing. Subsequently a single multi-gene construct that included all of the three human genes in the same plasmid was generated and expressed in seeds. Expression of the components of the human M6P elaboration machinery, including PT-αβ, PT-γ and the UCE within our high-producing-IDUA *Arabidopsis* cgl seed line (line A4.7; [6]) ensured an easier method to screen the resultant transgenic lines and showed that all three human proteins were successfully expressed simultaneously in the host cells. The purified IDUA derived from the *in planta* phosphorylation strategy was assessed for uptake into IDUA-deficient cells and for its ability to reduce pathological GAG substrates.

## 2. Materials and Methods

### 2.1. Gene Constructs to Generate the Human M6P-Elaborating Enzymes in cgl Seeds

A seed-specific expression cassette was used to direct the synthesis of the PT-αβ, PT-γ and UCE proteins in *Arabidopsis* cgl seeds. Specifically, a napin promoter, a napin signal peptide, and an arcelin 3′-terminator were used in the constructs. The human cDNAs specifying the PT-αβ, PT-γ and UCE were gifts from Dr. Stuart Kornfeld (Washington University School of Medicine, USA). The promoter, signal peptide, cDNA coding sequences (PT-αβ, PT-γ and UCE cDNAs), and terminator were fused together by an overlapping PCR approach and cloned into the binary vector pCambia1300 in *Escherichia coli* DH5α cells. All constructs were verified by DNA sequencing.

For the PT-αβ constructs, the αβ-precursor needs to be cleaved into separated α and β subunits after its synthesis. Plant cells possess similar but not identical processing enzymes to those of human cells. To ensure a greater possibility of correct cleavage, we designed three separate PT-αβ constructs each carrying one of the three different cleavage sites: the native S1P cleavage site, a furin site, and a 3 x kexin site.

To generate the human UCE protein as a soluble secreted protein for in vitro processing of IDUA, only the functional luminal domain of UCE was used, whereas the transmembrane domain and cytoplasmic tail were replaced with a HA-tag. Therefore in total, four constructs were engineered for UCE expression to generate both soluble and membrane-bound (Golgi-localized) forms. Since it is unknown whether the human UCE propeptide may be needed for functional expression/synthesis of the UCE in the plant host, the constructs for UCE expression either contained sequences specifying the propeptide or omitted those sequences.

Three multi-gene constructs were generated to simultaneously express the genes encoding PT-αβ, PT-γ, and UCE. Each multi-gene construct contained the *PT-αβ* gene carrying the S1P, furin or 3 x kexin cleavage sites. Each gene was placed in a separate expression cassette so that the expression and synthesis of each component would be controlled independently.

### 2.2. Transformation of Arabidopsis cgl-C5 Plants Transgenic for IDUA

The vectors were introduced into *Agrobacterium* LBA4404 and positive strains were used for transformation of *Arabidopsis thaliana* cgl-C5 plants transgenic for IDUA (the high-yielding IDUA line, A4.7, [6]) using the floral-dip method [28]. Seeds were harvested and screened on half-strength Murashige and Skoog (MS) solid medium in petri-dishes supplied with 20 mg/L of hygromycin. Putative transgenic seedlings were transferred to soil and grown in a plant growth chamber (21 °C, 16 h photoperiod). Mature seeds were harvested for further analysis.

### 2.3. Western Blot Analysis to Detect Components of M6P-Elaborating Machinery and IDUA in Transgenic Seed Extracts

For detection of the foreign proteins within total seed extracts, eighty micrograms of total seed protein of each sample were used. For detection of the purified plant-recombinant soluble UCE, ~50 ng of protein were used. Extracts were fractionated on a 10% SDS-PAGE gel and then electro-blotted onto a Hybond-P PVDF membrane (Amersham Biosciences, Piscataway, NJ, USA). Following a brief rinse in PBST (phosphate buffered saline containing 0.05% Tween-20), membranes were blocked for 1 h with 5% skimmed milk powder in PBST and then incubated with primary antibodies, which had been diluted in PBST and 3% skimmed milk powder, at 4 °C overnight. The primary antibodies included: anti-UCE antibody at 1:500 (Abcam, Toronto, Canada); anti-IDUA antibody at 1:1000 (Abcam, Toronto, Canada); anti-PTαβ antibody at 1:500 (kindly provided by Dr. Stuart Kornfeld); anti-PT-γ antibody at 1:500 (Abcam, Toronto, Canada). The blots were then washed three times in PBST (each 10 min) and incubated 3 h in secondary antibody (goat anti-rabbit IgG conjugated to horseradish peroxidase, 1:5000) (Thermo Fisher Scientific, Waltham, MA, USA). Following three washes (10 min each) in PBST, immunodetection was achieved by incubating with chemiluminescent reagent (ECL Advance Western Blotting Detection Kit, Amersham BioSciences), and detecting the signals by a LAS-4000 Luminescent Image Analyzer (Fujifilm Corporation, Tokyo, Japan).

### 2.4. Purification of Plant-Recombinant Soluble UCE and the UCE Activity Assay

To facilitate purification of the soluble UCE, a sequence specifying a HA-tag was added at the 3′ end of the UCE coding region. Total protein of seeds was extracted using a buffer containing 25 mM Tris-HCl, pH 7.5, 150 mM NaCl, 1% NP-40, 1 mM EDTA, 5% Glycerol, 0.05% Tween-20, and protease inhibitor cocktail (Sigma-Aldrich, St. Louis, MO, USA) at 1:100 dilution. Crude extracts were centrifuged at top speed for 10 min at 4 °C and the supernatant was used for UCE purification with anti-HA magnetic beads (Santa Cruz Biotechnology Inc., Dallas, TX, USA) according to the manufacturer’s instructions. After stringent wash steps, the UCE proteins were eluted with a solution containing 2 mg/mL of HA peptides in TBS-T buffer to preserve the stability and activity of the UCE enzyme. The HA peptide was custom synthesized from Applied Biological Materials (ABM, Richmond, BC, Canada).

The purified UCE enzyme was analyzed by the UDP-Glo™ Glycosyltransferase Assay Kit (Promega, Fitchburg, WI, USA) using UDP-GlcNac as the substrate according to the manufacturer’s instructions. Control reactions were performed without adding the enzyme or the substrate.

### 2.5. Efficacy of Purified Soluble UCE Enzyme to Remove GlcNAc from an In-Vitro-PT-Modified Plant-IDUA

To examine whether the soluble plant-recombinant UCE would be functional in M6P elaboration, the purified UCE was tested in an in vitro sequential processing of the plant-produced IDUA which possesses high-mannose N-glycans. The IDUA was first purified from cgl seeds and then processed with the recombinant soluble α_2_β_2_ GlcNAc-1-phosphotransferase [16], a gift from Dr. Stuart Kornfeld. Sixty μg purified cgl-IDUA and 1.5 μg soluble α_2_β_2_ GlcNAc-1-phosphotransferase were incubated at 37 °C for 1 h in a buffer (50 μL) containing 50 mM Tris-HCl (pH 7.4), 10 mM MgCl_2_, 10 mM MnCl_2_, 75 μM UDP-GlcNAc, 2 mg/mL bovine serum albumin and 1 mM ATP as described previously [4,12].

The products were transferred to a 4 mL Amicon 30 kDa centrifuge filter (Millipore Corp., Bedford, MA, USA) to exchange a buffer with 80 mM MES (pH 6.5) containing 0.5% Triton X-100. Subsequently the phosphorylated cgl-IDUA (IDUA-P-GlcNAc) was incubated with the plant-recombinant soluble UCE (~0.1 μg) at 37 °C in the above buffer for 1.5 h. A positive control was carried out side-by-side using a CHO-cell-derived recombinant soluble human UCE enzyme [27], a gift from Dr. Stuart Kornfeld. A negative control for the plant UCE was set up by not adding the phosphotransferase in the first step.

A small portion of cgl-IDUA is modified during the in vitro sequential processing. Since M6P-tagged lysosomal enzymes can be recognized by the cation-dependent M6P receptor (CD-MPR) [29], an affinity purification step was developed to remove the fraction of cgl-IDUA that was unmodified (i.e., did not contain a M6P tag). This utilized the recombinant human M6P receptor (ProSpec-Tany TechnoGene Ltd., Israel) cross-linked to Affi-gel 10 (Bio-Rad Laboratories Inc., Mississauga, ON, Canada) according to the manufacturer’s instructions. The in vitro processed protein mixture was added to recombinant M6P receptor-conjugated beads after a buffer exchange with the receptor binding buffer (20 mM MES, pH 6.5, 0.5 M NaCl, 1 mM MnCl_2,_ and 1 mM MgCl_2_) and incubated at 4 °C for 1 h. The beads were washed four times with 20 mM Tris-HCl (pH 7.0) containing 0.5 M NaCl, 1 mM MnCl_2_ and eluted with buffer comprised of 20 mM Tris-HCl, pH 7.0, 0.5 M NaCl, 1 mM EDTA, and 5 mM M6P (Sigma-Aldrich, St. Louis, MO, USA). Following the procedure, the IDUA activity was measured (as described in 2.8) and the treatment versus controls was assessed.

### 2.6. Purification of Recombinant Human IDUA from cgl Seeds

Recombinant IDUA proteins were purified from *Arabidopsis* cgl seeds, including cgl seeds expressing IDUA alone and representative seed lines simultaneously synthesizing all the four proteins (the PT-αβ, PT-γ, UCE, and IDUA). The protein purification protocol used Con-A Sepharose and anti-IDUA affinity chromatography and was carried out essentially as described [3].

### 2.7. Mice and Culture of Murine Fibroblasts

Idua-deficient mice (previously described in [30,31]) were obtained from the Jackson Laboratory (Bar Harbor, ME, USA) and kindly provided by Dr. Thorsten Schinke (Department of Osteology and Biomechanics, University Medical Center Hamburg-Eppendorf, Germany). Offspring from heterozygous matings were genotyped by PCR with the primers 5′-GGA ACT TTG AGA CTT GGA ATG AAC CAG-3′ and 5′-GAT TGT AAA TAG GGG TAT CCT GGA ATG AAC CAG-3′ and 5′-GGA TTG GGA AGA GAA TAG CAG GCA TGC T-3′ to amplify a 550 bp (wild-type allele) and/or a 350 bp fragment (mutant allele). Mice were housed in a pathogen-free animal facility at the University Medical Center Hamburg-Eppendorf (Germany), and experimental procedures were performed according to the institutional guidelines.

Isolation and cultivation of embryonic fibroblasts from wild-type and idua-deficient mice were performed as previously described [32].

### 2.8. IDUA Uptake Assays on Murine Fibroblasts

To monitor cellular uptake of recombinant IDUAs, 5 µg/mL Aldurazyme^®^, pIDUA (plant recombinant IDUA possessing high-mannose N-glycans) or M6P-pIDUA (plant recombinant IDUA putatively possessing M6P) were added to the medium for 4 h in the absence or presence of 20 mM M6P and/or 80 mM mannose (both from Sigma-Aldrich). Cells were lysed in 10 mM PBS (pH 7.4) containing 0.5% Triton X-100 and protease inhibitors for 30 min at 4 °C. After centrifugation at 10,000× *g*, supernatants were used for measurement of the protein content by the Roti^®^quant Protein Assay (Roth, Karlsruhe, Germany). To measure IDUA activity in cell extracts, 0.5 mM 4-methylumbelliferyl α-L-iduronate (Sigma-Aldrich, St. Louis, MO, USA) was used as a substrate in 100 mM Na-citrate (pH 4.6), 0.1% Triton X-100 and 150 mM NaCl. The incubation was stopped by addition of 0.4 M glycine/NaOH buffer (pH 10.4) after 2 h and the liberated 4-methylumbelliferone was measured at 355 nm (excitation) and 460 nm (emission).

### 2.9. Glycosaminoglycan Analysis

Embryonic fibroblasts from wild-type and Idua-deficient mice were incubated for 24 h with serum-free Opti-MEM™ medium containing 100 µCi/mL Na_2_^35^SO_4_ (Hartmann Analytic, Braunschweig, Germany). Cells were then washed twice with PBS and incubated for 24 h with serum-free Opti-MEM™ medium in the presence or absence of 5 μg/mL Aldurazyme^®^, pIDUA or “putative” M6P-pIDUA (hereafter in most places of the text referred to simply as M6P-pIDUA). To remove cell surface proteoglycans, cells were washed with PBS and treated with 0.05% trypsin/EDTA solution for 20 min. Afterwards the cells were lysed in 0.1 M NaOH and neutralized in 50 mM sodium acetate (pH 6.0) and 0.2 M NaCl. For protein digestion the lysates were incubated overnight with 100 μg/mL pronase (Sigma-Aldrich, St. Louis, MO, USA) at 37 °C. GAGs were purified using diethylaminoethylcellulose-sepharose anion exchange chromatography (Sigma-Aldrich, St. Louis, MO, USA) and the radioactive GAG amount was measured in a scintillation counter and related to the protein content.

## 3. Results

### 3.1. General Strategies for M6P Elaboration of Plant IDUA

**Figure 1:**
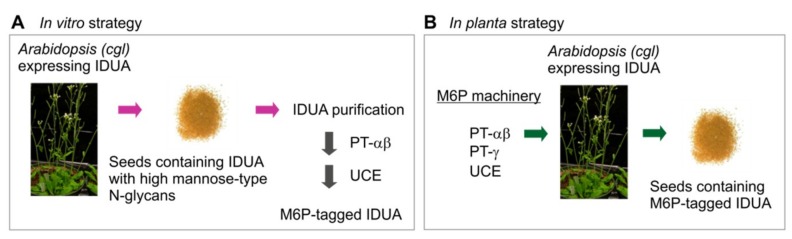
Strategies for in vitro (**A**) and *in planta* (**B**) elaboration of the M6P motif onto recombinant IDUA or other lysosome enzymes. The in vitro approach (A) requires purification of IDUA possessing high-mannose N-glycans from the transgenic plant host (cgl seeds); this is then subjected to sequential processing using purified recombinant soluble enzymes as reagents―the PT-α_2_β_2_ and the UCE. In the *in planta* strategy (B) the entire M6P elaborating enzymatic machinery components are simultaneously expressed in the same line that is already expressing the target hydrolase IDUA, so that the process of adding M6P to target proteins is essentially the same as that occurring in mammalian cells. PT: GlcNAc-1-phosphotransferase; IDUA: α-L-iduronidase.

Our plant-based platform—cgl seeds of *Arabidopsis*—Offers considerable advantages for the cost-effective production of recombinant lysosomal enzymes for use in ERT. For example, our elite cgl-line produces recombinant human IDUA at >5.7% total soluble protein. A possible strategy to modify plant recombinant IDUA with the M6P tag―the motif required for the lysosomal trafficking of many recombinant enzymes used in ERTs―is to treat a purified plant recombinant IDUA with a recombinant soluble PT α_2_β_2_ in vitro, lacking its transmembrane domains [4,6,16]), followed by treatment with a recombinant soluble UCE (Figure 1A). This approach has the drawback of requiring reagents which may be costly to generate and/or are available in only small quantities. Furthermore, the V_max_ of the transfer of GlcNAc-phosphate to plant recombinant IDUA in vitro is relatively low, although the K_m_ values reflect that the PT binds with great tenacity to the plant-recombinant target hydrolase [6,33].

An alternative strategy is to express the entire human M6P machinery in plants that are already expressing IDUA or another lysosome enzyme (Figure 1B). In this strategy, the entire M6P elaboration process will be carried out *in planta* via essentially the same mechanisms as in mammalian cells. Since recombinant proteins expressed in seeds are generally stable when the transgenic mature dry seeds are stored under conventional (cool and dry) conditions, e.g., iduronidase [34], the final recombinant products can be expected to remain stable. The resultant lysosome enzymes need only be purified when required.

### 3.2. Efficacy of Soluble Plant-Recombinant UCE in the In Vitro Sequential (PT-UCE) Processing of Purified cgl-IDUA

**Figure 2:**
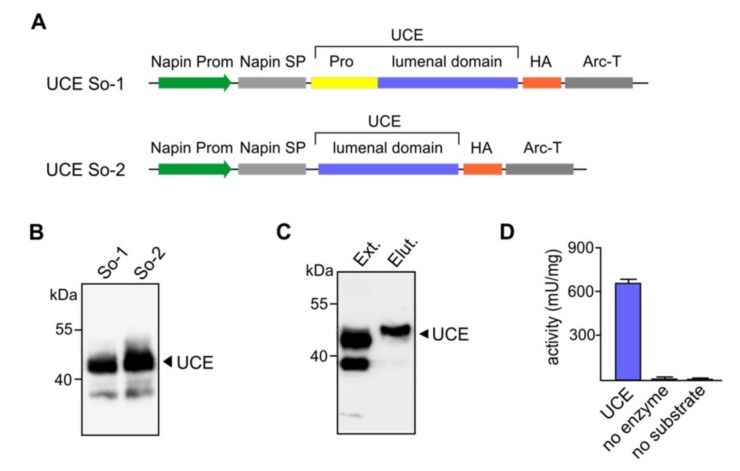
Expression of human UCE in *Arabidopsis* cgl seeds as a soluble secreted protein. (**A**) Diagrammatic representations of the constructs So-1 and So-2 for soluble/secreted UCE. Both constructs are under the control of the napin gene promoter (Prom). The So-1 construct consists the plant specific napin signal peptide (SP) in place of the native UCE signal peptide, the propeptide (Pro) and the lumenal domain. The transmembrane domain is replaced with a HA-tag. The transcription-termination sequence was derived from the arcelin gene (Arc-T). In the So-2 construct the UCE propeptide has been removed so that the UCE will not require cleavage (i.e., it will be constitutively active). (**B**) Representative Western blots show the synthesis of a prominent ~43-kDa polypeptide as predicted for So-1 and So-2 and indicate that the propeptide is correctly cleaved (So-1). (**C**) Purification of soluble UCE proteins from *Arabidopsis* cgl seeds. To facilitate the purification of the soluble secreted UCE proteins anti-HA conjugated beads were used. The UCE proteins were eluted with a HA-peptide solution to ensure that the native form and activities were preserved. Ext: Extract, Elut: eluted proteins. (**D**) For the UCE activity assay, the UDP-Glo™ Glycosyltransferase Assay kit was used to analyze the activities of the purified UCE using UDP-GlcNAc as the substrate. Control reactions, no enzyme, or no substrate addition were carried out in parallel. UCE: N-acetylglucosamine-1-phosphodiester α-N-acetylglucosaminidase.

Although our main objective is to conduct *in planta* phosphorylation, we wanted to first determine whether a plant-derived purified UCE would perform well under in vitro conditions on an appropriate IDUA substrate. Previously, a soluble UCE was generated in CHO cells, by removing the transmembrane domain and cytoplasmic tail to yield a secreted protein [23]. This modified recombinant protein could be readily purified and used in the sequential in vitro processing of lysosomal hydrolase targets. Therefore, we reasoned that a plant-derived soluble/secreted UCE could be of use for determining the fidelity of UCE function to remove the GlcNAc residue from substrate targets in the plant host, assuming that the secreted form would mimic a plant Golgi-localized UCE in terms of its catalytic characteristics.

ln mammalian cells the TGN-localized UCE occurs as a tetrameric transmembrane protein and functions to remove a covering GlcNAc from the M6P recognition marker on lysosomal acid hydrolases. The UCE is synthesized as an inactive proenzyme that is activated by the endoprotease furin, which cleaves an RARLPR↓D sequence to release a 24-amino acid propiece. As furin is localized in the TGN, newly synthesized UCE is inactive until it reaches this terminal compartment of the Golgi apparatus [27]. It was unknown if the propeptide would be essential for the functional expression, synthesis and folding of the UCE in the plant host cells. Since plant cells do not produce furin but other furin-like enzymes, it was equally unknown whether the UCE would be correctly processed in the same way as in the TGN of mammalian cells so that the propeptide piece would be removed. Therefore, to assess these possibilities, constructs with or without sequences specifying the propeptide were generated (Figure 2A). In place of the transmembrane domain and cytoplasmic tail sequences, the sequence encoding a HA-tag at the C-terminus of the soluble UCE was included in each construct to facilitate downstream purification of the enzyme.

Several dozens of transgenic plants were obtained for each of the UCE constructs to generate the soluble/secreted UCE protein. The representative Western blots show that the transgenic seed lines synthesize the 43-kDa soluble UCE protein of the expected molecular mass, and that the propeptide was correctly cleaved post-translationally (Figure 2B).

### 3.3. Purified Soluble UCE Enzyme Exhibited High Activities

To examine if the plant-soluble UCE is biologically active, the UCE was purified exploiting the HA-tag attached to its C-terminus. Total crude protein extracts from transgenic *Arabidopsis* cgl seeds were incubated with anti-HA conjugated beads. The UCE proteins attached to the beads were subsequently eluted with a HA-peptide solution to ensure that the native form and activity of the enzyme were preserved. Western blot analysis visualized the purified soluble UCE protein as a homogeneous single band of ~43 kDa (Figure 2C). The purified UCE appears to migrate slightly differently than the UCE within the crude protein extract. This might be because the protein is proportionally far less in amount in the crude extract. Alternatively, it may be because the crude extract contains detergent (1% NP-40), while the purified preparation does not. However, the purified UCE enzyme exhibited high specific activities while the bioluminescent signal in the two control reactions was close to zero (Figure 2D). The results clearly demonstrate that the UCE protein had been processed correctly after synthesis in the plant cell and retained its biological activity.

### 3.4. Purified Soluble UCE is Functional in Sequential in-vitro Phosphorylation of IDUA

To further test if the plant-produced UCE was able to function in the process of protein phosphorylation, the purified soluble UCE was used as the second step in an in vitro M6P elaboration assay in which plant-recombinant IDUA possessing high-mannose N-glycans served as the target protein. After cgl-IDUA was treated with recombinant soluble PT-α_2_β_2_ [6], the products were incubated with the plant-produced soluble UCE. The CHO cell-produced soluble UCE served as a positive control. A negative control for the plant-produced UCE excluded the soluble PT in the first step.

**Figure 3:**
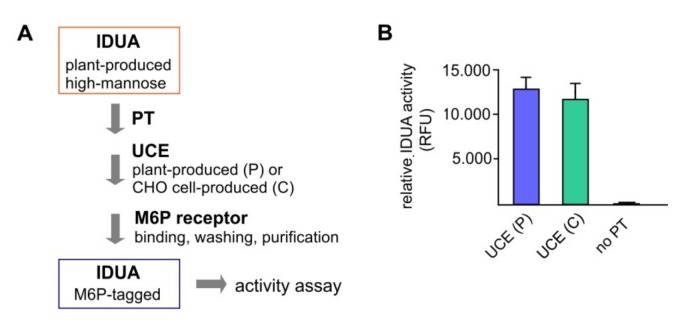
In vitro elaboration of M6P onto plant-produced IDUA using soluble UCE as a reagent. Two-step elaboration of M6P onto plant-produced purified IDUA was carried out using sequentially a soluble PT and a soluble UCE (the latter was either the plant-produced soluble UCE, or the CHO cell produced soluble UCE, as a control). (**A**) Procedure of M6P elaboration. After sequential treatment with the soluble PT and the soluble UCE, IDUA was applied to M6P receptor-conjugated beads and underwent stringent washing before elution. Control reaction without the PT was also carried out. (**B**) Relative activities of recovered IDUA after purification with M6P receptors (RFU: relative fluorescent unit).

The processed IDUA was applied to cation-dependent M6P receptor-conjugated beads and the eluted M6P-IDUA was used for activity analysis (Figure 3A). The relative activities of IDUA that had been treated with the plant UCE versus the CHO cell-derived UCE were equivalent, while the negative control (no PT-treatment) showed only little IDUA activity (Figure 3B). This indicates that all the non-M6P IDUA had been washed away from the M6P receptor-conjugated beads. The results demonstrated that the plant-produced soluble UCE functions correctly in the M6P elaboration process.

### 3.5. Expression of Individual Components of M6P-Elaboration Machinery in Arabidopsis cgl Seeds

**Figure 4:**
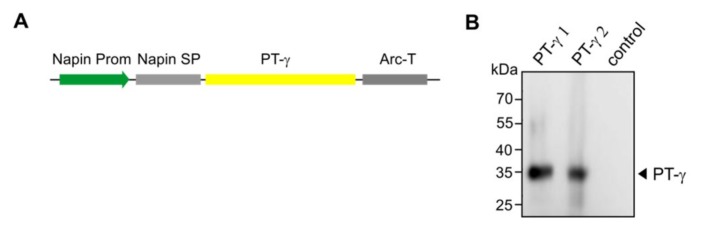
Expression of the PT-**γ** in *Arabidopsis* cgl seeds (IDUA-line A4.7). (**A**) A seed-specific napin promoter (Prom) was used to drive the expression of the human gene. The native PT-γ signal peptide was replaced with the plant-specific napin signal peptide (SP). The transcription-termination sequence was derived from the arcelin gene (*Arc-T*). (**B**) The Western blot shows representative lines that express the PT-γ protein in seeds using antibodies against the PT-γ subunit. As a control protein sample from the A4.7 seeds without the PT-γ construct was used.

Our main strategy is to devise the means for *in planta* M6P elaboration of lysosomal hydrolase targets (Figure 1B). In order to determine if the various components of the M6P machinery would be successfully synthesized in *Arabidopsis* seeds, we expressed the PT-γ, PT-αβ and UCE individually in cgl seeds of *Arabidopsis* (A4.7 line) which is high-yielding for IDUA. The expression of all of the genes was driven by the seed-specific napin promoter, and sequences for the napin signal peptide replaced those specifying the human signal peptides (Figure 4A).

After *Agrobacterium*-mediated transformation of *Arabidopsis*, performed individually for each construct, mature seeds were harvested and screened with the selectable marker. Putative transgenic plants were grown in a growth chamber to generate more seeds for Western blot analyses. Representative lines successfully expressed the PT-γ subunit as a polypeptide of the expected size (35 kDa) in considerable abundance (Figure 4B).

**Figure 5:**
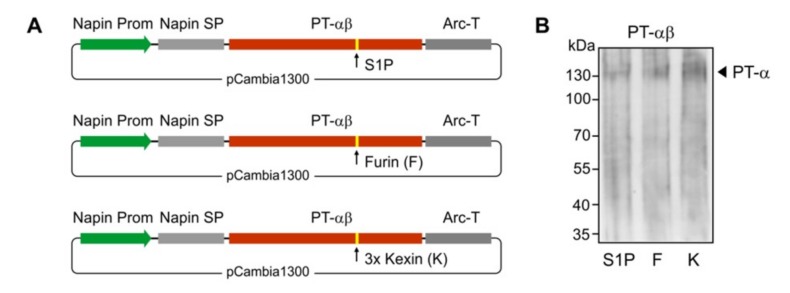
Expression of the PT-αβ in *Arabidopsis* cgl seeds (IDUA-line A4.7). (**A**) A seed-specific napin gene promoter (Prom) was used to drive the expression of the human genes. The native PT-αβ signal peptide was replaced with the plant-specific napin signal peptide (SP). Three options of cleavage sites were used/engineered and included the native S1P site, a furin (F) site and a 3 x kexin (K) site. The transcription-termination sequence was derived from the arcelin gene (*Arc-T*). (**B**) The Western blot shows the expression of the cleaved PT α subunit in the transgenic cgl line, each containing the sequence specifying either the S1P site (S1P), or furin site (F), or 3 x kexin (K) sites, as indicated.

The PT α and β subunits are encoded by a single gene in humans. For the expression of functional PT-αβ subunits, cleavage to generate the two subunits is a critical step but it was unknown if the plant cells would be able to perform this cleavage. To maximize success rate, we engineered three different cleavage sites in the constructs between the sequences specifying the two subunits (Figure 5A). The S1P site is the native sequence in human cells. Furin cleavage sites occur in other human Golgi-resident proteins (e.g., within the UCE, between the propeptide and the luminal domain). Finally a 3 x Kexin cleavage site was engineered, as this recognition site is efficiently utilized by plant host Golgi proteases. The three constructs were individually used to transform *Arabidopsis*, and the transgenic cgl seeds were generated. As shown by Western blot analyses of total protein seed extracts from representative transgenic lines, all the three cleavage options allowed successful processing of the αβ-precurosr subunits as demonstrated by the presence of a polypeptide of ~130 kDa corresponding to the cleaved α subunit (Figure 5B).

**Figure 6:**
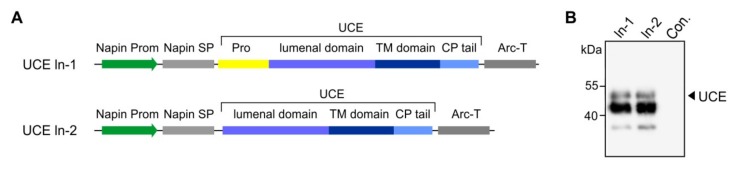
Expression of human UCE in *Arabidopsis* cgl seeds as a TGN-localized protein. (**A**) Diagrammatic representations of the TGN-localized UCE In-1 and In-2 constructs. Both contain the napin gene promoter (Prom) to drive seed-specific expression, the plant-specific napin signal peptide (SP) in place of the native UCE signal peptide, the transmembrane (TM) and cytoplasmic (CP) tail of UCE. The transcription-termination sequence was derived from the arcelin gene (*Arc-T*). In construct ln-1 the propeptide is intact, whereas the ln-2 construct has the sequences specifying the propeptide removed to produce a constitutively active UCE. (**B**) Representative Western blots show the synthesis of a prominent ~50 kDa polypeptide as predicted for In-1 and In-2, and again show that the propeptide is correctly cleaved (In-1). A prominent lower molecular weight 43 kDa polypeptide is also detected. Con.: non-transgenic control.

As in the case of the two soluble UCE proteins (So-1 and So-2), several dozens of lines were screened for expression of the In1 (with propeptide) and In2 (without propeptide) constructs for generating the UCE as an integral protein localized within the plant TGN (Figure 6A). The ~50-kDa band is indicative of the mature full size UCE protein and further indicates that the UCE-propeptide precursor underwent cleavage of the propeptide within the plant host cells (Figure 6B).

All of the transgenic *Arabidopsis* cgl lines individually expressing PT-γ, PT-αβ, and UCE showed normal growth characteristics and did not exhibit any obvious stress phenotypes. This indicated that none of the proteins is toxic to the plant cells and that the growth and development of the transgenic plants are not adversely affected.

### 3.6. Simultaneous Expression of M6P Elaboration Machinery Components in IDUA-Line

**Figure 7:**
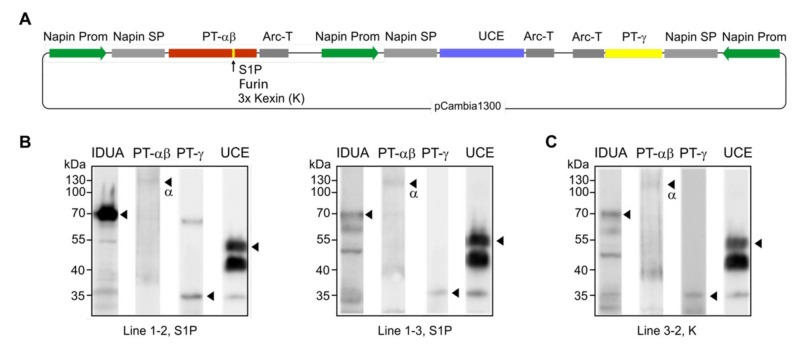
Simultaneous expression of the entire M6P enzymatic machinery including PT-αβ, PT-γ, and UCE in *Arabidopsis* cgl seeds (IDUA-line A4.7). (**A**) In the construct design, note that the expression of each protein is independently controlled by separate promoters and terminators. Since it was unknown if and how plants would cleave the human PT-αβ protein, the native S1P site, a furin site and a 3 x kexin site were engineered in separate constructs. (**B**,**C**) Western blots of representative lines show that all four proteins are expressed in the seeds. Lines 1–2 and 1–3 (**B**) are expressing the PT-αβ construct containing the S1P cleavage site while line 3–2 (**C**) has a 3 x kexin site in the PT-αβ construct. No line for the furin site construct was obtained with seeds expressing all four proteins.

*In planta* elaboration of the M6P-tag onto IDUA proteins requires that all three genes encoding the M6P-machinery and the target hydrolase IDUA are expressed simultaneously (Figure 1B). Considering the three options for PT-αβ cleavage, the three different constructs to effect simultaneous expression were used to individually transform the *Arabidopsis* cgl line A4.7 [6] that was already synthesizing a high level of human IDUA. Although each gene is controlled independently for more efficient transcription, a single plasmid encoding PT-αβ, PT-γ, and UCE was generated to make the screening process much easier (Figure 7A). Several dozens of transgenic plants were obtained from the transformation. Western blots of seed extracts from three representative lines revealed that all four proteins are synthesized at detectable levels (Figure 7B,C). Seeds of lines with high levels of IDUA, such as line 1–2 (Figure 7B), are ideal candidates for subsequent examination of the status of IDUA phosphorylation and efficacy.

### 3.7. Assessment of Therapeutic Efficacy: Cellular Uptake and GAG Reduction

**Figure 8:**
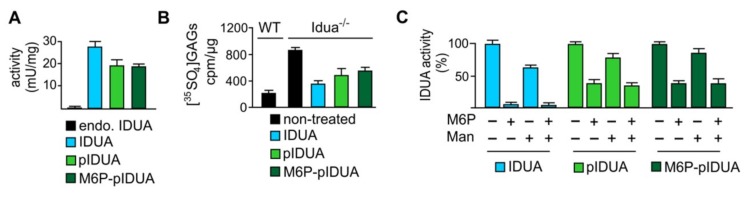
Uptake of plant-derived IDUA in primary fibroblasts and clearance of intracellular GAG storage. (**A**) Specific IDUA activity in wild-type embryonic fibroblasts incubated for 4 h in the absence (endo., endogenous) and presence of CHO cell-derived, M6P-tagged IDUA (Aldurazyme^®^), plant-derived, high mannose-type IDUA with no M6P (pIDUA), and plant-IDUA generated *in planta* with M6P-elaborating machinery (putative M6P-pIDUA). (**B**) [^35^SO_4_]GAG content in wild-type (WT) and Idua^−/−^ cells after 24 h pulse and 24 h chase in the presence of IDUA (Aldurazyme^®^), pIDUA, and putative M6P-pIDUA as indicated. Non-treated cells were used as controls. (**C**) Relative IDUA activity in wild-type embryonic fibroblasts incubated for 4 h in the presence of IDUA (Aldurazyme^®^), pIDUA, and putative M6P-pIDUA as well as 20 mM M6P and 80 mM mannose (Man), as indicated.

To assess the therapeutic efficacy, uptake studies using purified “putative” M6P-IDUA generated *in planta* (i.e., IDUA proteins derived from seeds of line 1–2) were performed on cultured idua-deficient mouse embryonic fibroblasts. The recombinant lysosomal enzyme putatively containing the M6P motif was efficiently taken up by the cells (Figure 8A). Its extent of uptake based on internal IDUA activity was similar to that of the cgl-IDUA possessing high-mannose N-glycans, but somewhat less than the commercial counterpart Aldurazyme^®^. Idua deficiency results in the intracellular accumulation of non-degraded GAGs [30]. Accordingly, at baseline the [^35^SO_4_]GAG content after a 24 h pulse period with Na_2_^35^SO_4_ (100 µCi/mL) and a 24 h chase period was about five-fold higher in idua^-/-^ cells compared to wild-type controls (Figure 8B). The plant M6P-IDUA led to a substantial reduction of GAGs by about 44%, as compared to Aldurazyme^®^, which effected a reduction of ~56% (Figure 8B). However, the extent of GAG substrate reduction effected by the purified plant enzyme putatively possessing the M6P tag was no greater than that displayed by the plant-derived mannose-terminated IDUA, suggesting poor efficiency of modification of IDUA with the M6P tag. This may have been due to an instability of the PT *in planta*, since the PT transcripts are present in multiple lines but the proteins corresponding to the αβ subunits are present only in low amounts.

The uptake of Aldurazyme^®^ in wild-type fibroblasts was almost completely inhibited by M6P but only partially by mannose demonstrating that the endocytosis of IDUA is mainly mediated by M6P receptors (Figure 8C). In contrast, the characteristics of inhibition by M6P and/or mannose were similar for the two plant IDUAs, which both showed approximately a 60% reduction of uptake by M6P and about a 20% reduction of uptake in the presence of mannose. Additionally, in contrast to the commercial enzyme, both of the plant enzymes (pIDUA and putative M6P-pIDUA) still showed considerable uptake in the presence of both inhibitors suggesting the existence of alternative transport pathways that are independent of the conventional routes via mannose receptor- and M6P receptor-mediated mechanisms.

## 4. Discussion

Most lysosomal enzymes including IDUA are thought to require a M6P tag for efficient uptake and lysosomal delivery in human cells. In mammalian cells, the two Golgi-localized enzymes PT and UCE act sequentially to elaborate M6P tags on lysosomal hydrolases. The PT residing in the *cis*-Golgi apparatus catalyzes the adding of GlcNAc-P from UDP-GlcNAc to selected terminal mannose residues of the target lysosomal enzyme’s high mannose N-glycans. Subsequently, the TGN-localized UCE hydrolyzes the bond between the anomeric carbon of the GlcNAc and the oxygen of the phosphate group to liberate the GlcNAc and expose the phosphate [10].

Plant-recombinant IDUA derived from *Arabidopsis* cgl seeds is biologically active, but does not possess the M6P motif because the plant host cells do not have the required enzymatic machinery—i.e., the PT and UCE. In order to develop the plant-produced IDUA further for ERT, one strategy is to elaborate the M6P tag in vitro, i.e., when IDUA purified from the plant host is sequentially processed by soluble forms of the PT and UCE [2,3,15]. Because of the drawbacks of in vitro processing, our main strategy is to engineer *Arabidopsis* lines so that they simultaneously express the human M6P enzymatic machinery (i.e., PT and UCE) together with IDUA for in vivo M6P elaboration. If the endogenous recombinant plant IDUA can be modified *in planta*, the only downstream processing that would be required is the purification of the M6P-IDUA proteins.

Our first step was to express a soluble/secreted form of the UCE in the *Arabidosis* cgl seed host. In doing so we sought to address the following questions: (1) is the propeptide sequence needed for correct synthesis/folding of the UCE? Further, does the plant cell host have an appropriate protease to cleave the propeptide, thus creating an active enzyme in the late Golgi? (2) Does the purified plant-produced soluble UCE exhibit its normal biological activity, i.e., can it cleave the terminal GlcNAc from an artificial substrate? Further, is the purified UCE functional in the sequential in vitro processing of cgl-purified IDUA to elaborate the M6P-motif onto its high-mannose N-glycans? Our results answered these questions and provided evidence that the Golgi-targeted UCE should be functional both in vivo and *in planta* in processing its lysosomal hydrolase target. The soluble UCE is expected to be secreted from the cgl cells, since during its transit through the TGN the propeptide was presumably cleaved by a plant enzyme similar to the mammalian furin protease.

For our *in planta* strategy for M6P elaboration of IDUA we have developed a cgl line that is very high-yielding for IDUA (A4.7), accumulating the human protein in seeds to ~5.7% total soluble protein [3]. Not only is the seed platform attractive from the point of view of the high yield of recombinant IDUA, but the seeds represent a stable repository for the recombinant IDUA protein. For example, the activity of IDUA is preserved for a prolonged period (2 months and greater) when the mature dry transgenic seeds are stored under conventional conditions (4 °C) [34]. In addition, when multiple foreign proteins are expressed within the vegetative tissues of plants, often normal growth and development is disrupted and/or the plant exhibits severe stress responses. However, when expression is exclusive to seeds, these negative effects are typically minimized. Indeed in the present case, simultaneous synthesis of the four human proteins in the same line did not result in visible stress phenotypes, and the growth and development of the transgenic plants were normal.

Expression of all of the genes was driven by a seed storage protein promoter (napin). The most significant drawback was the relatively low level of accumulation of the PT-αβ subunits. The most likely reason for this could be the instability of the synthesized proteins *in planta*, since the transcripts were present in multiple lines but the proteins corresponding to the αβ subunits were found only in low amounts. Another less likely reason could be that the antibodies used for Western blot analyses (specific for the PT-α subunit) did not effectively detect the plant-produced proteins. Nonetheless, detection of the PT-α subunit, albeit in low amount, revealed that the plant host cells possess proteases that are capable of cleaving the human S1P and furin sites, as well as the plant specific 3 x kexin site between the α and β subunits. Unlike PT-αβ, accumulation of the PT-γ and UCE proteins was reasonably high in the cgl seeds as robust amounts of the polypeptides were detected by Western blot analyses.

When synthesized as an integral TGN-localized protein within the plant host cells, the UCE appeared to be correctly processed since its propeptide was removed. A “mature” 50-kDa protein is predicted for this *in planta* form (excluding the propeptide), and this species was present in considerable abundance. Interestingly, a ~43 kDa polypeptide was also present, and in greater abundance. This is likely a processed form indicating that a portion of the UCE is transiting out of the TGN to the plant cell surface. Supporting this idea, in mammalian cells the UCE resides in the TGN and also cycles between the TGN and plasma membrane [24].

It was important to ensure correct cleavage of the propeptide of the TGN-localized UCE within the plant host as the enzyme would otherwise be inactive. In mammalian cells, the cleavage is effected by a furin protease as soon as the protein reaches the TGN [27]. There is no homolog of the furin enzyme in plants; still, a systemin-binding protein found in tomato has similar functions to furin [35]. There might also be other unknown enzymes in plants with functions equivalent to furin.

Since the various enzymes act in concert to affect the M6P modification process, even if a small proportion of the synthesized proteins are functional, they should be sufficiently active to process the target IDUA produced in the same cells. Toward assessing therapeutic efficacy, we purified the IDUA from representative transgenic plant lines that simultaneously expressed all of the M6P-elaborating enzymatic machinery. We then compared the plant IDUA putatively possessing the M6P tag (M6P-pIDUA) for its ability to be taken up by wild-type and Idua-deficient primary cultured fibroblasts as compared to the commercial IDUA (Aldurazyme^®^). As a control we assessed IDUA purified from cgl seeds which possesses predominantly high mannose N-glycans (>94%). The putative M6P-pIDUA was efficiently taken up by the fibroblasts; its extent of uptake was about 70% of that exhibited by Aldurazyme^®^. Moreover, the M6P-pIDUA led to a substantial reduction of GAGs by about 44%, as compared to its commercial counterpart which effected a reduction of ~56%. However, the extent of both uptake of recombinant enzyme and GAG substrate reduction effected by the plant M6P-IDUA was no greater than that displayed by the plant-derived mannose-terminated IDUA. Several reasons could lead to this. First, because the purified IDUA is comprised of a mixture of high-mannose-terminated IDUA and putative M6P-IDUA from the transgenic seeds, it is very possible that the proportion of the M6P-IDUA protein is relatively small―too small to make a difference in the therapeutic characteristics of the enzyme. Further, the PT-αβ subunits may not have been correctly localized to the *cis*-Golgi of plant cells, leading to instability of the PT-α/β or of the PT heterohexamer and/or loss of its biological function. The PT-α, -β and -γ subunits may also be unable to assemble into the α_2_β_2_ γ_2_ holoenzyme in the plant cells. Because the M6P elaboration process itself is complex as are the steps required for synthesis, processing and targeting of the components of the machinery, there could be several other underlying reasons for the relative inefficiency of the process. Remaining challenges include boosting the *in planta* production of the PT α and β subunits, and following that, undertaking studies to characterize the M6P-containing oligosaccharides on our therapeutic enzyme, as well as determine the actual proportion of M6P-IDUA produced. However, if substantially greater numbers of transgenic plants are screened, it is entirely possible to find lines that produce sufficient amounts of M6P-tagged IDUA to alter the therapeutic characteristics of the population of IDUA molecules.

The results of lysosomal hydrolase uptake using primary mammalian Idua-deficient fibroblasts very interestingly show that despite the M6P receptor being the predominant plasma membrane receptor type in these cells [36], the plant IDUA, which is mannose-terminated, nonetheless shows significant uptake into the cells and moreover is effective in reducing GAG substrate. Other groups are likewise finding that mannose-terminated lysosomal hydrolases exhibit a considerable extent of therapeutic efficacy for Gaucher disease and Niemann–Pick disease [37,38]. Furthermore, mannose-dependent uptake of commercial M6P-tagged arylsulfatase B (Naglazyme^®^ (BioMarin Pharmaceutical Inc., San Rafael, CA, USA) was shown in primary cultured cells from MPS VI mice [39]. These studies have opened up the possibility that a plant-based platform for generating mannose-terminated enzyme will possess efficacy in ERT regimes, i.e., without the requirement for additional processing to create the M6P motif. A moss-derived mannose-terminated version of acid α-galactosidase A (largely Man3), was recently evaluated as a potential Fabry disease ERT by examining its uptake into disease fibroblasts and by substrate clearance in tissues of the Fabry disease mouse model [40]. In the mouse model, the moss α-galactosidase A displays similar tissue distribution and enzyme half-life to that of the traditional M6P-tagged α-galactosidase. Thus M6P targeting is not the only means by which lysosomal enzymes can be taken up by cells. In fact, restricting enzyme replacement strategies to solely utilizing the M6P receptor pathway may ultimately be limiting.

## 5. Conclusions

In conclusion, the experiments in this report showed that it is generally feasible to express the entire human M6P-machinery enzymes in plants simultaneously along with the target lysosomal hydrolase (such as IDUA) toward performing the phosphorylation process *in planta*. However, there is a clear need to assess the actual proportion of M6P-IDUA that is produced. Certainly an *in planta* approach would have significant advantages over in vitro strategies to effect M6P elaboration for ERT applications. Even if improvements in the *in planta* M6P-tagging process could not be achieved, an exciting outcome of testing the plant-derived mannose-terminated IDUA was that this form of the enzyme yielded results comparable to those obtained with the commercial IDUA (Aldurazyme^®^), and a significant amount of the plant-IDUA is trafficked by an M6P receptor-independent pathway. Thus, a plant-based platform for generating lysosomal enzymes, even without the M6P motif, may represent an alternative and cost-effective strategy to the conventional ERT.
